# PPARs modulate cardiac metabolism and mitochondrial function in diabetes

**DOI:** 10.1186/s12929-016-0309-5

**Published:** 2017-01-10

**Authors:** Ting-Wei Lee, Kuan-Jen Bai, Ting-I Lee, Tze-Fan Chao, Yu-Hsun Kao, Yi-Jen Chen

**Affiliations:** 1 Graduate Institute of Clinical Medicine, College of Medicine, Taipei Medical University, 250 Wu-Xing Street, Taipei, 11031 Taiwan; 2 Division of Endocrinology and Metabolism, Department of Internal Medicine, Wan Fang Hospital, Taipei Medical University, Taipei, Taiwan; 3 Division of Pulmonary Medicine, Department of Internal Medicine, Wan Fang Hospital, Taipei Medical University, Taipei, Taiwan; 4 School of Respiratory Therapy, College of Medicine, Taipei Medical University, Taipei, Taiwan; 5 Department of General Medicine, School of Medicine, College of Medicine, Taipei Medical University, Taipei, Taiwan; 6 Division of Cardiology, Department of Medicine, Taipei Veterans General Hospital, Taipei, Taiwan; 7 Institute of Clinical Medicine, and Cardiovascular Research Center, National Yang-Ming University, Taipei, Taiwan; 8 Department of Medical Education and Research, Wan Fang Hospital, Taipei Medical University, Taipei, Taiwan; 9 Division of Cardiovascular Medicine, Department of Internal Medicine, Wan Fang Hospital, Taipei Medical University, Taipei, Taiwan

**Keywords:** Cardiomyopathy, Diabetes mellitus, Mitochondria, Metabolism, Peroxisome proliferator-activated receptors

## Abstract

Diabetic cardiomyopathy is a major complication of diabetes mellitus (DM). Currently, effective treatments for diabetic cardiomyopathy are limited. The pathophysiology of diabetic cardiomyopathy is complex, whereas mitochondrial dysfunction plays a vital role in the genesis of diabetic cardiomyopathy. Metabolic regulation targeting mitochondrial dysfunction is expected to be a reasonable strategy for treating diabetic cardiomyopathy. Peroxisome proliferator-activated receptors (PPARs) are master executors in regulating glucose and lipid homeostasis and also modulate mitochondrial function. However, synthetic PPAR agonists used for treating hyperlipidemia and DM have shown controversial effects on cardiovascular regulation. This article reviews our updated understanding of the beneficial and detrimental effects of PPARs on mitochondria in diabetic hearts.

## Background

Diabetes mellitus (DM) is one of the most common chronic diseases, and its prevalence continues to increase worldwide [1, 2]. Cardiovascular disease is the leading cause of morbidity and mortality in patients with DM. Diabetic cardiomyopathy is recognized as a distinct disease entity, since diabetic patients have an increased incidence of heart failure in the absence of hypertension, coronary artery disease, or valvular heart disease [3–5]. Diabetic cardiomyopathy is characterized by cardiac lipid accumulation, myocardial fibrosis, and increased myocardial cell death, all of which lead to left ventricular remodeling and hypertrophy, diastolic dysfunction, and ultimately systolic impairment [6]. The pathophysiology of diabetic cardiomyopathy is complex and yet to be fully elucidated. Altered cardiac metabolism and mitochondrial dysfunction are proposed mechanisms underling diabetic cardiomyopathy [7].

Peroxisome proliferator-activated receptors (PPARs) are nuclear hormone receptors and major executors of modulating glucose and lipid homeostasis [8]. There are three PPAR isoforms (PPAR-α, PPAR-β/PPAR-δ, and PPAP-γ), which differ in distribution, function, and ligand specificity. Accumulating evidence suggests that PPARs play crucial roles in cardiovascular disease [9]. PPAR isoforms are differentially expressed in the atria and ventricles of diabetic hearts because of the increased inflammatory cytokines and oxidative stress [10]. Moreover, we found increases in protein and messenger (m) RNA expressions of PPAR-γ, but decreases in protein and mRNA expressions of PPAR-α and PPAR-δ in hypertensive hearts. Diabetic spontaneously hypertensive rats were associated with greater reductions in cardiac PPAR-α and PPAR-δ, but higher increases in PPAR-γ mRNA and protein levels than were spontaneously hypertensive rats [11]. Diabetic cardiomyopathy is associated with an increase in cardiac PPAR-γ and a decrease in PPAR-α, resulting in altered glucose transportation, increased cardiac lipid accumulation, and progressive diabetic cardiomyopathy [7, 10–12]. Calcitriol and histone deacetylase inhibitor improved diabetic cardiomyopathy by modulating cardiac PPAR expressions and regulating fatty acid metabolism [13, 14]. Mitochondria are the center of fatty acid and glucose metabolism and are thus likely to be impacted by metabolic derangements in DM. Proper mitochondrial function is critical for maintaining optimal cardiac performance. Several mouse models of mitochondrial defects are relevant to human cardiomyopathy [15]. Patients with inherited mitochondrial disorders frequently manifest cardiac dysfunction, such as dilated or hypertrophic cardiomyopathy and conduction defects [16]. This review elucidates our current understanding of different PPARs and their agonists on mitochondrial function in diabetic hearts.

## Cardiac metabolism in normal and diabetic hearts

Fatty acids and glucose are principle substrates for myocardial energy metabolism. Under physiological conditions, fatty acid β-oxidation constitutes the major energy source in the heart. In contrast, glycolysis predominates during pathological stimuli, such as during ischemia and heart failure [17, 18]. The cardiac oxygen consumption for adenosine triphosphate (ATP) production is greater when utilizing fatty acids than when using glucose. However, there is increased fatty acid β-oxidation and reduced glucose oxidation in diabetic hearts. The increased fatty acid utilization in diabetic hearts is associated with reduced cardiac efficiency, which is a hallmark of diabetic cardiomyopathy [19–21]. Diabetic hearts are more vulnerable to ischemic injury due to their constrained fuel substrate flexibility. In DM, high circulating lipid levels increase fatty acid delivery to cardiomyocytes due to insulin resistance. Cardiac glucose uptake is mainly controlled by insulin-mediated recruitment of glucose transporter type four (GLUT4) from the intracellular compartment to plasma membranes. High fatty acid concentrations in diabetic hearts may impair insulin signal transduction, thereby decreasing GLUT4 translocation and reducing glucose uptake [19]. In contrast, expressions of fatty acid transporters are increased in diabetic hearts. The enhanced cluster of differentiation 36 (CD36) and fatty acid-binding proteins can promote fatty acid uptake, and increased fatty acids activate PPAR-α, which facilitates cardiac fatty acid metabolism. Activation of cardiac PPAR-α not only increases expressions of genes involved in fatty acid β-oxidation but also suppresses glucose utilization [19, 20]. Myocardial fatty acid uptake and oxidation are increased, and glucose uptake and oxidation are reciprocally suppressed in mice with cardiac-specific overexpression of PPAR-α, which exhibited cardiac dysfunction that mimics diabetic cardiomyopathy [22]. Moreover, augmented fatty acid β-oxidation causes accumulation of citrate in the cytosol. High concentrations of citrate inhibit the action of phosphofructokinase 1 (the rate-limiting enzyme) in glycolysis. Pyruvate, the product of glycolysis, is transported to mitochondria and decarboxylated to acetyl-CoA by pyruvate dehydrogenase. Both increased fatty acid β-oxidation and PPAR-α activation lead to suppression of pyruvate dehydrogenase, which impairs glucose oxidation [19]. Our previous study found that diabetic hearts expressed more fatty acid transporters and metabolic enzymes, including CD36, carnitine palmitoyltransferase 1 (CPT-1), and phosphorylated acetyl CoA carboxylase. In addition, diabetic cardiomyopathy is associated with activation of enzymes controlling the formation of triglycerides, such as diacylglycerol acyltransferase (DGAT) [13, 14]. The shuttling of excessive fatty acids into triglyceride synthesis serves to minimize the generation of toxic lipid metabolites. However, chronic metabolic derangement results in cardiac lipid accumulation and produces diabetic cardiomyopathy. Alternations of cardiac metabolism in DM are summarized in Table 1.

**Table 1:** Altered cardiac metabolism in diabetes

Alteration of cardiac metabolism	Mechanism
Changes in fuel preference [19–21]	Increases fatty acid β-oxidation
	Decreases glucose oxidation
Defect in glucose utilization [19, 20, 22]	Reduces GLUT4 expression and translocation
	High fatty acid oxidation suppresses PFK1 through accumulation of citrate in the cytosol
	High fatty acid oxidation inhibits PDH through activation of PDK
Alterations in fatty acid utilization [13, 14, 19, 20]	Increases the expression of fatty acid transporters
	PPAR-α activation promotes the expressions of genes that regulate fatty acid β-oxidation

*GLUT4*, glucose transporter type 4; *PFK1*, phosphofructokinase 1; *PDH*, pyruvate dehydrogenase; *PDK*, pyruvate dehydrogenase kinase

## Mitochondrial dysfunction in diabetic hearts

Mitochondria act as the powerhouse of cells because they generate most of the cell’s supply of ATP. Cardiomyocytes contain a relatively large amount of mitochondria (approximately 40% of the cardiomyocyte volume is comprised of mitochondria), because the heart has a high and continuous demand for ATP [18]. In response to diverse physiological and nutritional conditions, it is critical to control the metabolic activity of mitochondria to meet cellular energy requirements.

A substantial body of evidence has demonstrated that there is significantly impaired mitochondrial function in diabetic cardiomyopathy. Excessive fatty acid uptake in diabetic hearts results in an altered mitochondrial architecture and reduced expressions of genes involved in mitochondrial oxidative phosphorylation [21]. Moreover, PPAR-α activates genes involved in fatty acid uptake and β-oxidation, but does not increase expressions of genes associated with the tricarboxylic acid cycle or mitochondrial oxidative phosphorylation. Thus, the upregulated mitochondrial fatty acid uptake and β-oxidation may exceed the capacity of downstream mitochondrial respiration and lead to an accumulation of toxic lipid metabolites, which further worsens insulin resistance. In addition, increased fatty acid β-oxidation augments delivery of electrons to the mitochondrial electron transport chain and results in an elevated mitochondrial inner membrane potential, which stimulates reactive oxygen species (ROS) generation [19, 20]. ROS directly impair mitochondria and/or oxidize lipids to yield reactive lipid peroxidation, as a result of inducing oxidative damage to mitochondrial proteins that are associated with energy metabolism and oxidative phosphorylation. Moreover, ROS can activate mitochondrial uncoupling, and subsequently reduce cardiac efficiency [19–21, 23]. Excessive fatty acids lead to the generation of ceramide. Ceramide triggers apoptosis through nitric oxide- and peroxynitrite-mediated opening of mitochondrial permeability transition pores and release of cytochrome c. Ceramide also suppresses mitochondrial respiration through directly inhibiting the activity of mitochondrial electron transport chain complex III [19, 24]. Incomplete fat oxidation and accumulated toxic fatty acid intermediates lead to mitochondrial dysfunction through hyperpolarization of the mitochondrial inner membrane potential, mitochondrial uncoupling, and generation of ROS [21, 23, 25].

Upregulation of mitochondrial uncoupling proteins is another potential explanation for the reduced mitochondrial efficiency in diabetic hearts. Uncoupling proteins cause proton leaks across mitochondrial membranes from ATP synthesis, thereby decreasing the generation of mitochondrial superoxide. Increased mitochondrial uncoupling is presumably an adaptive mechanism; however, sustained activation of mitochondrial uncoupling may adversely affect cardiomyocyte ATP production and contractile function in DM [21, 25, 26]. Mitochondrial calcium handling was proposed to represent a mechanism for coordinating the ATP supply and demand for cardiomyocyte contractions [27]. Mitochondrial calcium uptake may also act as a spatial buffering system, which regulates the activity of calcium-dependent processes and signaling [28]. The mitochondrial transmembrane potential is not only required for ATP synthesis, but also plays a crucial role in driving calcium accumulation in mitochondria. Disruption of the mitochondrial membrane potential in the diabetic heart leads to altered mitochondrial calcium handling which contributes to the development of diabetic cardiomyopathy [21].

Mitochondrial DNA encodes proteins for the electron transport chain, which is localized in the mitochondrial inner membrane and drives ATP production through oxidative phosphorylation. The damage to mitochondrial DNA leads to impairment of mitochondrial respiration and ATP synthesis. Because dysfunctional mitochondria are a major source of ROS production, mitochondrial DNA is a vulnerable target of ROS damage [23, 29]. Several investigations have implied that cardiomyocyte apoptosis promotes the development of diabetic cardiomyopathy. Diabetic mice showed enhanced apoptotic signaling in the heart that was associated with changes in the mitochondrial membrane potential and the opening in mitochondrial permeability transition pores [30]. Findings from mitochondrial proteomic studies in diabetic hearts supported the role of mitochondrial-induced apoptosis in diabetic cardiomyopathy [31]. Furthermore, cardiac fibrosis is a major feature of diabetic cardiomyopathy. Apoptotic cardiomyocytes are replaced by fibrotic tissues. Myocardial fibrosis contributes to increased stiffness and decreased compliance of the ventricular wall, resulting in left ventricle dysfunction. Mitochondrial dysfunction augments ROS production, which is thought to be a crucial driving force for cardiac fibrosis [6, 32–34].

A number of studies provided evidence for mitochondrial alternations in hearts of patients with DM. Diastolic dysfunction in association with a reduction in myocardial energy metabolism was demonstrated using magnetic resonance techniques in asymptomatic normotensive patients with well—controlled DM [35]. Mitochondria in atrial tissues of diabetic patients revealed a sharply decreased capacity for respiration and increased mitochondrial hydrogen peroxide emissions, suggesting an increase in oxidative stress [36]. An association of worsened cardiac mitochondrial respiration with a reduced mitochondrial calcium retention capacity with decreased contractile performance in heart tissues of diabetic patients was shown before the onset of clinical cardiomyopathy [37].

## PPARs regulate myocardial energy metabolism in diabetes

**Fig. 1:**
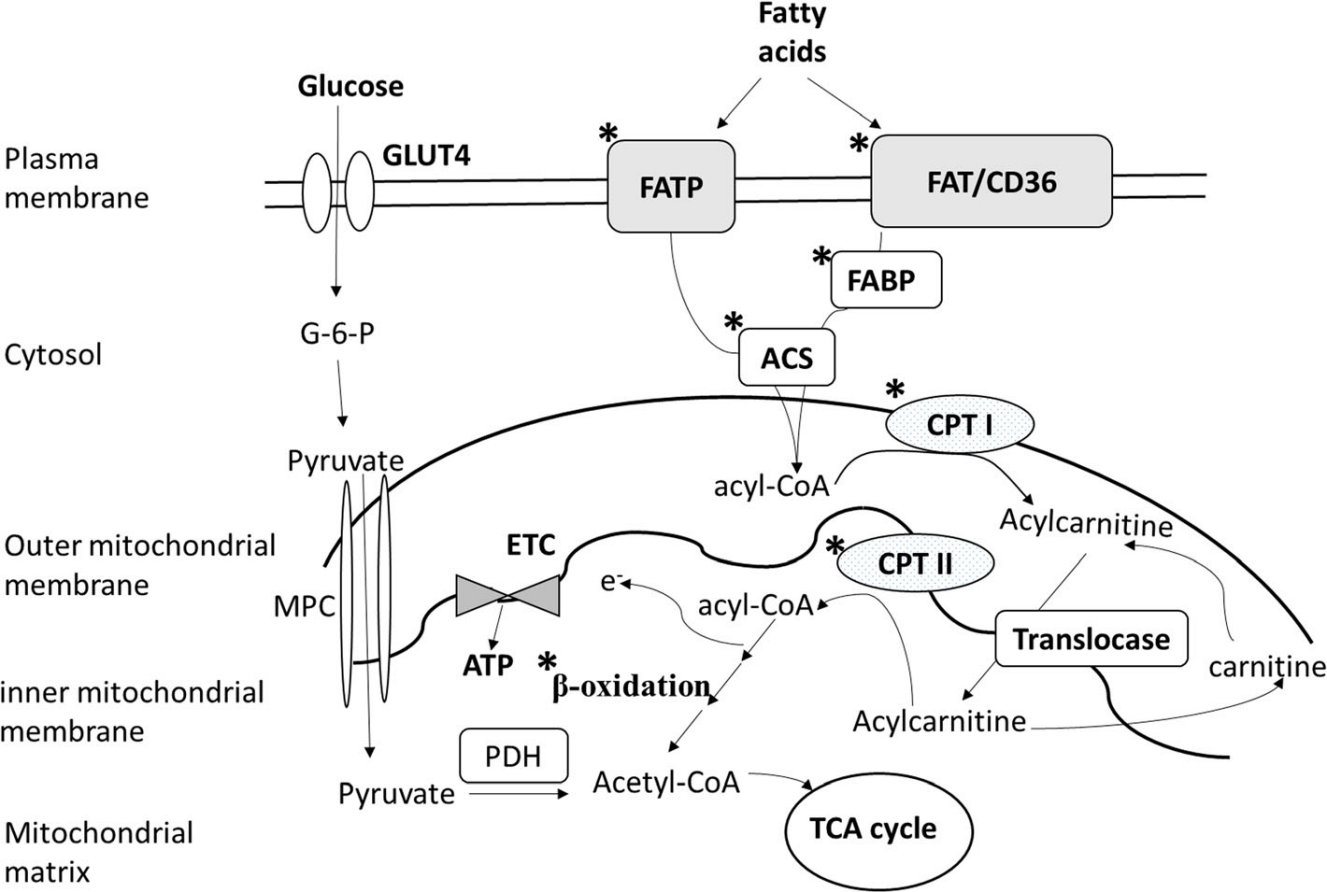
Peroxisome proliferator-activated receptor (PPAR)-α regulates fatty acid utilization and β-oxidation in cardiac metabolism. Stars indicate transporters and enzymes involved in fatty acid metabolism which are regulated by PPAR-α. FATP, fatty acid transport protein; FABP, fatty acid binding protein; ACS, acyl-CoA synthetase; CPT-I, carnitine palmitoyltransferase-I; CPT-II, carnitine palmitoyltransferase II; TCA, tricarboxylic acid; GLUT4, glucose transporter four; MPC, mitochondrial pyruvate carrier; and PDH, pyruvate dehydrogenase. Modified from [8]

PPAR-α was first cloned in 1990 and so named because it was activated by the lipid-lowering drug, fibrate, which causes hepatic peroxisome proliferation in rodents [38]. PPAR-α is the principal regulator modulating energy and lipid homeostasis through transcriptional regulation of fatty acid metabolic enzymes. PPAR-α is abundantly expressed in tissues with a high capacity for mitochondrial fatty acid oxidation, such as the liver and heart. Figure 1 shows that PPAR-α regulates lipid metabolism by controlling expressions of enzymes that are directly involved in fatty acid uptake (CD36), triglyceride synthesis (DGAT), and β-oxidation (CPT-1, acyl-CoA dehydrogenase) [12, 21, 25]. Several studies indicated that diabetic hearts were associated with increased expression of PPAR-α because of high levels of circulating fatty acids [25]. However, our previous study demonstrated a significant decline in PPAR-α and an increase in PPAR-γ protein levels in diabetic hearts despite an increase in cardiac fatty acid oxidation. These findings indicated that hyperglycemia is associated with a compensatory response for preserving the contractile function through activation of inflammatory cytokines [39]. Mouse models lacking PPAR-α were protected against the development of diabetes-induced cardiac hypertrophy. In contrast, transgenic overexpression of PPAR-α in diabetic hearts displayed severe cardiomyopathy and was accompanied by myocardial triglyceride accumulation [40, 41].

PPAR-δ is expressed in multiple tissues and shares certain similarities with PPAR-α in regulating cardiac lipid metabolism. Cardiac-specific deletion of PPAR-δ down regulates constitutive myocardial fatty acid oxidation, and induces myocardial lipid accumulation and cardiac hypertrophy in mice [42]. Fatty acids and PPAR-δ-selective ligands increase fatty acid oxidation through transcriptional activation in both neonatal and adult cardiomyocytes [43, 44]. However, the PPAR-δ-selective ligand does not modify the expression of PPAR-α in cultured cardiomyocytes. PPAR-δ activation can partially restore the blunted expressions of genes encoding cardiac fatty acid oxidation enzymes in PPAR-α-knockout mice. These findings suggest that PPAR-δ-regulated cardiac fatty acid metabolism might not wholly interact with PPAR-α [44, 45]. Moreover, there was increasing myocardial glucose utilization without myocardial lipid accumulation or cardiac dysfunction in transgenic mice with cardiac-specific overexpression of PPAR-δ. Accordingly, PPAR-α and PPAR-δ may exert distinct cardiac metabolic regulatory actions [46].

PPAR-γ plays a crucial role in regulating lipid storage and adipogenesis. PPAR-γ is expressed at levels far below those of PPAR-α and PPAR-δ in the heart. PPAR-γ ligands do not affect the fatty acid oxidation rate or metabolic gene expression in cardiomyocytes [43]. It was suggested that PPAR-γ modulates cardiac energy metabolism through its effects on extra cardiac tissues. Activation of PPAR-γ promotes glucose uptake and triglyceride synthesis in adipose tissues. Reductions in circulating glucose and fatty acid levels caused by PPAR-γ activation may directly modulate cardiac PPAR-α and PPAR-δ activities [45]. Cardiac-specific PPAR-γ-overexpressing transgenic mice developed dilated cardiomyopathy with increased myocardial lipid and glycogen stores and upregulated cardiac expressions of genes associated with fatty acid utilization and glucose metabolism [47]. The mechanism underlying cardiomyopathy in PPAR-γ-overexpressing transgenic mice was hypothesized to be combined lipid and glucose toxicity [48].

Adenosine monophosphate-activated protein kinase (AMPK) and PPAR-γ co-activator (PGC)-1α are two major signaling molecules that regulate mitochondrial biogenesis. AMPK upregulates mitochondrial biogenesis through activation of PGC-1α, which is a master metabolic regulator that coordinates gene expressions in association with mitochondrial biogenesis and respiratory function [49]. Lee et al. showed that diabetic hearts have a lower ratio of phosphorylated AMPK2*α* to total AMPK2*α* and greater expression of PGC-1*α* compared to those of control rats [13, 14]. The up regulation of PGC-1α enables diabetic hearts to enhance their mitochondrial oxidative capacity [25]. Therefore, up regulation of PPAR-α and PGC-1α may initially be adaptive responses in diabetic hearts [21, 25, 50]. However, sustained increases in fatty acid β-oxidation are detrimental to cardiac mitochondria and further promote the development of diabetic cardiomyopathy [21, 23, 25].

## PPARs modulate mitochondrial function

### Effects of PPAR-α on mitochondria

Transgenic mice with cardiac-specific overexpression of PPAR-α had disorganized mitochondria, altered mitochondrial cristae density and architecture, and decreased expressions of genes involved in mitochondrial metabolism, including the tricarboxylic acid cycle and oxidative phosphorylation [51]. The cristae of mitochondria increased in number and density in cardiomyocytes of PPAR-α-null mice [52]. These findings suggest that abnormal expression of PPAR-α is linked to an altered mitochondrial structure and metabolic function.

Fibrates are synthetic PPAR-α agonists that are used as lipid-lowering agents. Several laboratory findings suggested that fibrates modulate mitochondrial function with potential beneficial or deleterious effects (Table 2). Ureido-fibrate-5 is a potent PPAR-α agonist and exerts a marked triglyceride-lowering effect by stimulating mitochondrial CPT-1-mediated fatty acid β-oxidation in both the liver and muscles [53]. In addition, fibrates also have an effect on glucose homeostasis. Fenofibrate improved insulin sensitivity not only by lowering serum lipid levels but also by enhancing mitochondrial fatty acid β-oxidation in skeletal muscles of fructose-fed rats [54]. Two weeks of fenofibrate treatment (5 mg/kg) ameliorated insulin resistance accompanied by an improved mitochondrial oxidative capacity in pediatric burn patients [55]. Mitochondrial oxidative stress was implicated in the pathogenesis of Batten disease, a rare and fatal autosomal recessive neurodegenerative disorder. Fenofibrate and gemfibrozil (1 μM) reduced mitochondrial membrane potential depolarization, thereby inhibiting the apoptosis of lymphoblast cells in Batten disease [56]. Pretreatment of female rats with gemfibrozil prior to global cerebral ischemia-reperfusion resulted in neuroprotection by modulating mitochondrial biogenesis and apoptosis [57]. Activation of PPAR-α with WY-14,643, an experimental ligand, or fenofibrate protects mice from acetaminophen-induced hepatotoxicity. This protective effect is mediated by up regulating the PPAR-α target gene that encodes mitochondrial uncoupling protein 2, which serves to prevent mitochondria from oxidative stress through decreasing the generation of mitochondrial ROS [58]. However, fibrates may cause mitochondrial dysfunction. A 24-h fenofibrate exposure (100 μM) impaired mitochondrial function in rat skeletal muscles through inhibiting the activity of mitochondrial respiratory chain complex I [59]. Gemfibrozil and WY-14,643 at toxicologically relevant concentrations altered mitochondrial bioenergetics through inducing the mitochondrial permeability transition which caused inhibition of oxidative phosphorylation and ATP synthesis in mitochondria in the rat liver [60]. Chronic treatment with WY-14,643 impaired myocardial contractile function while decreasing mitochondrial respiratory function and increasing mitochondrial uncoupling in rats [61].

**Table 2:** Effects of peroxisome proliferator-activated receptor (PPAR)-α agonists on mitochondria

PPAR-α agonists	Effects on mitochondria
Potential beneficial effects	
Fenofibrate [54, 55, 56, 58]	Stimulates mitochondrial fatty acid β-oxidation
	Improves mitochondrial oxidative capacity
	Reduces mitochondrial membrane potential depolarization and apoptosis
	Upregulates mitochondrial uncoupling protein 2
Gemfibrozil [57, 56]	Reduces mitochondrial membrane potential depolarization and apoptosis
	Modulates mitochondrial biogenesis and apoptosis
WY-14,643 [58]	Upregulates mitochondrial uncoupling protein two
Ureido-fibrate-5 [53]	Induces mitochondrial CPT I expression
	Stimulates mitochondrial fatty acid β-oxidation
Possible harmful effects	
Fenofibrate [59]	Inhibits mitochondrial respiratory chain complex I activity
WY-14,643, Gemfibrozil [60, 61]	Induces the mitochondrial permeability transition

*CPT I*, carnitine palmitoyltransferase I

### Effects of PPAR-γ on mitochondria

Overexpression of cardiac PPAR-γ via the cardiac α-myosin heavy chain promoter produced a distorted architecture of the mitochondrial inner matrix and disrupted cristae in PPAR-γ transgenic mice [47]. Transgenic mice overexpressing PPAR-γ2 had significantly increased expression of mitochondrial uncoupling protein one, elevated levels of PGC-1α, and reduced mitochondrial ATP concentrations in the subcutaneous fat [62]. Cardiac expression of the gene encoding manganese superoxide dismutase as a mitochondrial antioxidant was suppressed in cardiac-specific PPAR-γ-knockout mice [63].

Thiazolidinediones (TZDs) are synthetic PPAR-γ agonists and are used to treat DM. In addition to glucose metabolism, TZDs also exert several beneficial effects including lipid-lowering and anti-inflammation actions. However, troglitazone and rosiglitazone were respectively withdrawn from the market due to hepatotoxicity and increased cardiovascular risk. Our previous study showed that rosiglitazone can upregulate PPAR-γ mRNA and protein expressions, which might explain the harmful effects of the PPAR-γ agonist in DM given that PPAR-γ is already overexpressed in diabetic hearts [39]. In addition, we also found that rosiglitazone significantly changed cardiac calcium regulatory and electrophysiological characteristics, thereby enhancing arrhythmogenicity in DM with hypertension [64]. Numerous investigations have suggested that TZDs have important effects on mitochondrial function and biogenesis (Table 3). Expressions of genes in mitochondrial respiratory complexes I ~ IV were significantly down regulated in subcutaneous adipose tissues of diabetic patients and were restored in response to rosiglitazone treatment. Rosiglitazone also increased the relative amount of mitochondria in diabetic patients compared to control groups [65]. Pioglitazone treatment significantly increased the mitochondrial DNA copy number and expressions of factors involved in mitochondrial biogenesis and genes involved in the fatty acid oxidation pathway in adipocytes of patients with DM [66]. PPAR-γ also plays a crucial role in energy homeostasis observed in Huntington’s disease, which is characterized by mutant Huntingtin protein aggregates in the brain. Rosiglitazone protected a neuroblastoma cell line from a mutant Huntingtin protein-evoked mitochondrial deficiency [67]. Rosiglitazone can promote T lymphocyte survival by allowing cells to maintain the mitochondrial membrane potential following growth factor withdrawal or glucose restriction at doses that induce optimal PPAR-γ transcriptional activity. This suggests that PPAR-γ activation may potentially augment immune responses of diabetic patients [68]. However, TZDs demonstrated varying degrees of hepatotoxicity in an in vitro model, with troglitazone exhibiting the highest mitochondrial toxicity, followed by rosiglitazone and then pioglitazone. TZD-induced hepatotoxicity may involve alterations in mitochondrial respiratory function, changes in membrane permeability, and mitochondrial structural damage [69]. An in vitro study demonstrated that both rosiglitazone and pioglitazone at supra-physiological concentrations (100 μM) directly inhibited mitochondrial respiratory chain complex I activity and cell respiration in rat skeletal muscles [70]. In addition, PPAR-γ activation is associated with fluid retention, heart failure, and bone loss, thereby limiting the clinical use of TZDs.

**Table 3:** Effects of peroxisome proliferator-activated receptor (PPAR)-γ agonists on mitochondria

PPAR-γ agonists	Effects on mitochondria
Potential beneficial effects	
Rosiglitazone [65, 67, 68, 73, 74]	Upregulates expressions of mitochondrial respiratory complex I ~ IV genes
	Increases the relative number of mitochondria
	Maintains mitochondrial potential to promote cell survival
	Regulates mitochondrial pyruvate import
Pioglitazone [66, 73–75]	Increases the mitochondrial DNA copy number
	Increases mitochondrial biogenesis
	Increase genes involved in the fatty acid oxidation
	Regulates mitochondrial pyruvate import
	Controls maximal mitochondrial respiratory rates
Possible harmful effects	
Troglitazone, Rosiglitazone, and Pioglitazone [69, 70]	Alters mitochondrial respiratory function
	Changes membrane permeability
	Damages the mitochondrial structure
	Inhibits mitochondrial complex I activity and cell respiration

Substantial evidence has shown that TZDs exert direct and rapid PPAR-γ-independent effects on mitochondrial respiration, thereby leading to changes in glycolytic metabolism and fuel substrate specificity [71, 72]. It was shown that clinically relevant concentrations of TZDs acutely, specifically, and partially inhibit mitochondrial pyruvate carrier activity, thereby improving cellular glucose handling in human myocytes [73]. Laboratory studies revealed that TZDs have a recognition site in the inner mitochondrial membrane that is comprised of a protein complex, which is involved in mitochondrial pyruvate importation [74]. Pioglitazone was shown to specifically bind to a protein named mitoNEET, which is an iron-containing outer mitochondrial membrane protein, that is involved in controlling maximal mitochondrial respiratory rates [75]. Therefore, these findings suggest that development of novel molecules designed to maintain this mitochondrial interaction while specifically avoiding significant interactions with PPAR-γ is very appropriate for clinical treatments.

## Conclusions

Impaired mitochondrial biogenesis and function associated with derangement of cardiac metabolism play vital roles in the pathogenesis of diabetic cardiomyopathy. Therefore metabolic regulation targeting mitochondrial dysfunction may show therapeutic potential for treating diabetic cardiomyopathy. Synthetic PPAR-α and PPAR-γ agonists not only regulate expressions of genes involving lipid and glucose metabolism, but also modulate mitochondrial function and therefore appear to be promising treatments for diabetic cardiomyopathy. However, unfavorable effects of PPAR activation on cardiac mitochondria were also observed. Additional studies are required to develop optimal pharmacological approaches to improve mitochondrial function in diabetic hearts.

## Abbreviations

AMPK: Adenosine monophosphate-activated protein kinase
ATP: Adenosine triphosphate
CD36: Cluster of differentiation 36
CPT-1: Carnitine palmitoyltransferase 1
DGAT: Diacylglycerol acyltransferase
DM: Diabetes mellitus
GLUT4: Glucose transporter type 4
PGC-1α: PPAR-γ co-activator-1α
PPAR: Peroxisome proliferator-activated receptor
ROS: Reactive oxygen species
TZD: Thiazolidinedione
